# Dibenzo[*b*,*g*]indeno­[1′,2′:3,4]fluoreno[1,2-*d*]oxonine-5,11,16,21-tetra­one

**DOI:** 10.1107/S1600536812046363

**Published:** 2012-11-17

**Authors:** Shaaban Kamel Mohamed, Mehmet Akkurt, Muhammad N. Tahir, Antar A. Abdelhamid, Adel A. Marzouk

**Affiliations:** a Chemistry Department, Faculty of Science, Minia University, Egypt; b Chemistry and Environmental Division, Manchester Metropolitan University, Manchester M1 5GD, England; c Department of Physics, Faculty of Sciences, Erciyes University, 38039 Kayseri, Turkey; d University of Sargodha, Department of Physics, Sargodha, Pakistan; e Pharmaceutical Chemistry Department, Faculty of Pharmacy, Al Azhar University, Egypt

## Abstract

The asymmetric unit of the title compound, C_34_H_16_O_5_, contains two independent mol­ecules (*A* and *B*) with similar conformations. The two benzene rings attached to the nine-membered ring are inclined to one another at 63.62 (14)° in mol­ecule *A* and 68.23 (12)° in mol­ecule *B*. One intra­moleculer C—H⋯O hydrogen bond occurs in mol­ecule *A* and two are observed in mol­ecule *B*. In the crystal, mol­ecules are linked by weak C—H⋯O hydrogen bonds, forming a three-dimensional network structure with *R* ^2^_2_(10) and *R* ^2^_2_(24) ring motifs. Aromatic π–π stacking interactions [centroid–centroid distances = 3.7572 (19), 3.6996 (19) and 3.7043 (19) Å] are also observed. The unit cell contains a pair of voids of 37 (2) Å^3^ about an inversion centre but the residual electron density (highest peak = 0.19 e Å^−3^ and deepest hole = −0.20 e Å^−3^) in the difference Fourier map suggests that no solvent mol­ecule occupies this void.

## Related literature

1,3-Indandione undergoes self-condensation quite easily, see: Zargar & Khan (2012 ▶). For industrial and biological applications of indandion containing compounds see: Seniutinas *et al.* (2012 ▶); Jin *et al.* (2009 ▶). For ring conformations, see: Cremer & Pople (1975 ▶).
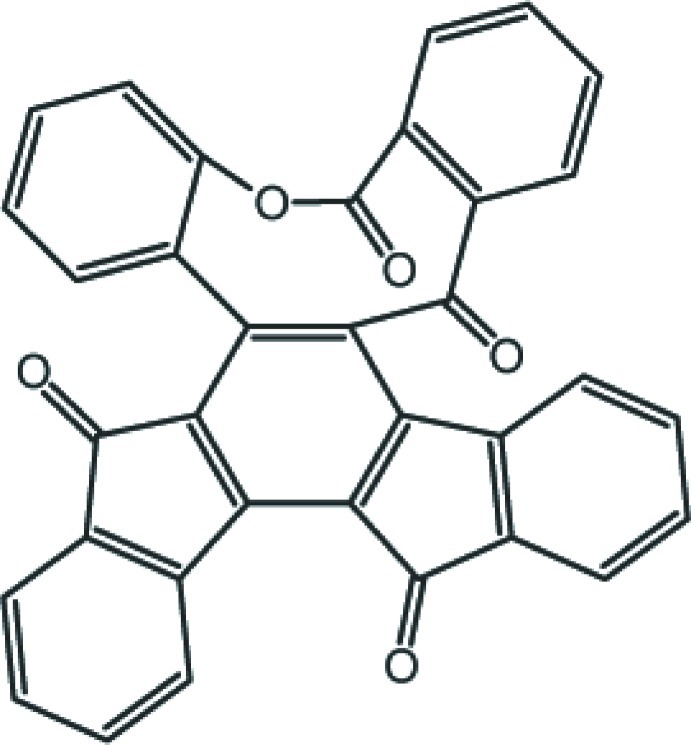


## Experimental

### 

#### Crystal data

C_34_H_16_O_5_
*M* *_r_* = 504.47
Triclinic, 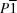

*a* = 12.230 (4) Å
*b* = 12.346 (3) Å
*c* = 16.794 (5) Å
α = 82.229 (7)°
β = 72.773 (6)°
γ = 81.181 (10)°
*V* = 2382.5 (12) Å^3^
*Z* = 4
Mo *K*α radiation
μ = 0.10 mm^−1^
*T* = 296 K
0.28 × 0.18 × 0.14 mm

#### Data collection

Bruker Kappa APEXII CCD diffractometer
Absorption correction: multi-scan (*SADABS*; Bruker, 2005 ▶) *T*_min_ = 0.980, *T*_max_ = 0.987
38137 measured reflections
10895 independent reflections
4858 reflections with *I* > 2σ(*I*)
*R*_int_ = 0.061

#### Refinement

*R*[*F* ^2^ > 2σ(*F* ^2^)] = 0.056
*wR*(*F* ^2^) = 0.135
*S* = 0.94
10895 reflections
704 parameters
H-atom parameters constrained
Δρ_max_ = 0.19 e Å^−3^
Δρ_min_ = −0.21 e Å^−3^

### 

Data collection: *APEX2* (Bruker, 2007 ▶); cell refinement: *SAINT* (Bruker, 2007 ▶); data reduction: *SAINT*; program(s) used to solve structure: *SIR97* (Altomare *et al.*, 1999 ▶); program(s) used to refine structure: *SHELXL97* (Sheldrick, 2008 ▶); molecular graphics: *ORTEP-3 for Windows* (Farrugia, 2012 ▶); software used to prepare material for publication: *WinGX* (Farrugia, 2012 ▶) and *PLATON* (Spek, 2009 ▶).

## Supplementary Material

Crystal structure: contains datablock(s) global, I. DOI: 10.1107/S1600536812046363/xu5643sup1.cif

Structure factors: contains datablock(s) I. DOI: 10.1107/S1600536812046363/xu5643Isup2.hkl

Supplementary material file. DOI: 10.1107/S1600536812046363/xu5643Isup3.cml

Additional supplementary materials:  crystallographic information; 3D view; checkCIF report

## Figures and Tables

**Table 1:** Hydrogen-bond geometry (Å, °)

*D*—H⋯*A*	*D*—H	H⋯*A*	*D*⋯*A*	*D*—H⋯*A*
C24—H24⋯O10^i^	0.93	2.56	3.302 (3)	137
C30—H30⋯O5^ii^	0.93	2.60	3.507 (3)	166
C32—H32⋯O7^iii^	0.93	2.49	3.398 (3)	166
C33—H33⋯O4	0.93	2.32	3.087 (3)	139
C39—H39⋯O1^iv^	0.93	2.36	3.115 (3)	139
C56—H56⋯O8	0.93	2.52	3.210 (3)	131
C58—H58⋯O7^v^	0.93	2.52	3.377 (3)	154
C67—H67⋯O9	0.93	2.36	3.117 (3)	139

Symmetry codes: (i) 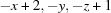; (ii) 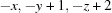; (iii) 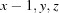; (iv) 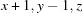; (v) 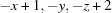.

**Fig. 1.**
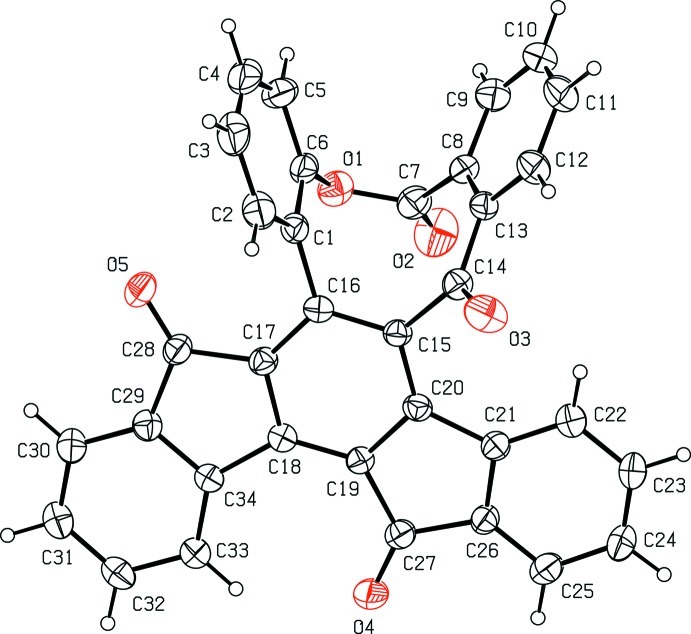
View of the molecule A of two molecules of (I) in the asymmetric unit. Displacement ellipsoids for non-H atoms are drawn at the 30% probability level.

**Fig. 2.**
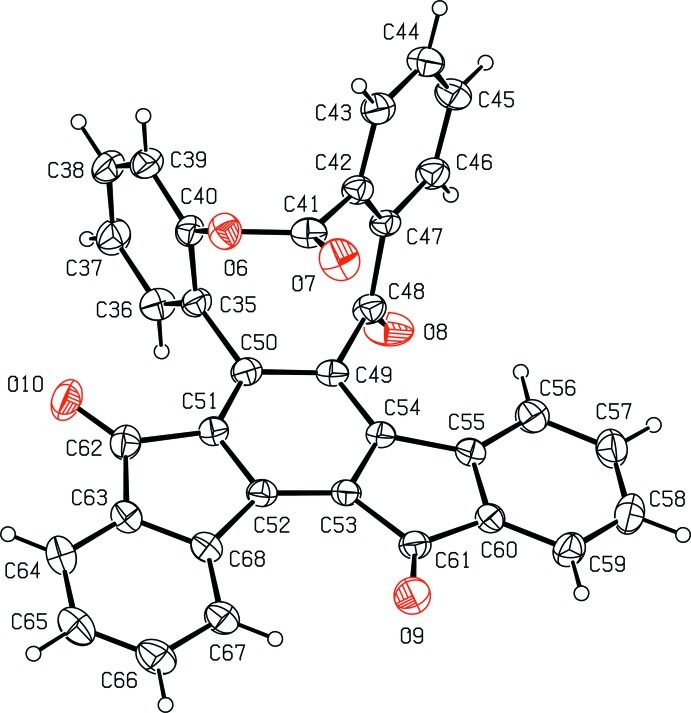
View of the molecule B of two molecules of (I) in the asymmetric unit. Displacement ellipsoids for non-H atoms are drawn at the 30% probability level.

**Fig. 3.**
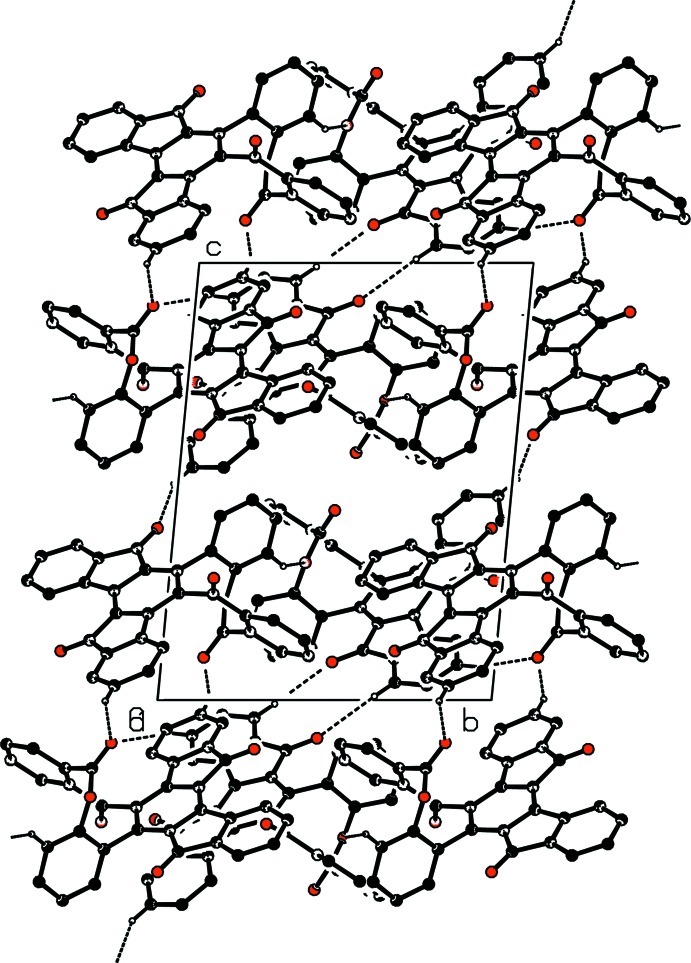
View of the molecular packing of (I) down the a axis. H atoms not involved in hydrogen bonding have been omitted for clarity.

## supplementary crystallographic information

### Comment

1,3-indandione is an important member of class of 1,3-diketo compounds. It is a very strong C-nucleophile. It undergoes self-condensation quite easily, resulting in a dimer *e.g.* bindone and trimer (Zargar & Khan, 2012). Due to such unusual chemical reactivity, 1,3-indandione showed a wide range of industrial and pharmaceutical applications. It is a founder structure in most of non linear optical (NLO) compounds, chromophores (Seniutinas *et al.*, 2012) and pesticide molecules such as diphacinone, chlorophacinone, valone, and Pindone (Jin *et al.*, 2009). Such facts prompted us to synthesize and study the crystal determination of the title compound (I).

Figures 1 and 2 show two enantiomeric molecules (A with O1 and B with O6) of the title compound in the asymmetric unit. The values of the geometric parameters of both molecules A and B are similar with each other. In molecule A, the two benzene rings (C1–C6 and C8–C13) which are attached to the nine-membered ring are inclined to one another by 63.62 (14)°, and in molecule B, the two benzene rings (C35–C40 and C42–C47) by 68.23 (12)°.

In molecule A, the puckering parameters for the nine-membered ring (O1/C1/C6—C8/C13—C16) are Q(2) = 1.880 (3) Å, φ (2) = 58.83 (8)°; Q(3) = 0.189 (2) Å, φ (3) = 10.2 (8)°; Q(4) = 0.118 (3) Å, φ (4) = 136.5 (12) ° and Q_T_ (total puckering amplitude) = 1.893 (3) Å (Cremer & Pople, 1975). In molecule B, the puckering parameters for the nine-membered ring (O6/C35/C40—C42/C47—C50) are Q(2) = 1.864 (3) Å, φ (2) = 246.61 (7) °; Q(3) = 0.223 (2) Å, φ (3) = 177.7 (7) °; Q(4) = 0.143 (2) Å, φ (4) = 313.4 (10) ° and Q_T_ (total puckering amplitude) = 1.882 (3) Å.

In the crystal, C—H···O hydrogen bonds link the A and B molecules (Fig. 3, Table 1). The crystal packing is stabilized by additional three π–π stacking interactions [*Cg*5···*Cg*31 (*x*, *y*, *z*) = 3.7572 (19) Å, *Cg*7···*Cg*32(-1 + *x*, *y*, *z*) = 3.6996 (19) Å and *Cg*31···*Cg*31(1 - *x*, -*y*, 2 - *z*) = 3.7043 (19) Å; where *Cg*5, *Cg*7, *Cg*31 and *Cg*32 are the centroids of the C15–C20, C29–C34, C55–C60 and C63–C68 rings, respectively].

### Experimental

The title compound was obtained from a reaction mixture of 4 mmol (576 mg) indandione and 2 mmol (244 mg) salicylaldehyde in presence of 1 mmol (90 mg) 1,3-diaminopropan-2-ol in 50 ml e thanol. The reaction mixture was refluxed for 6 h at 350 K. The excess solvent was evaporated under vacum and the obtained solid was recrystallized from acetic acid to afford a good yield (82%) of dark green crystals (m.p. 575 K) suitable for X-ray diffraction.

### Refinement

All H atoms were located geometrically and treated as riding atoms with C—H = 0.93 Å, and with *U*_iso_(H) = 1.2*U*_eq_(C).

### Crystal data

**Table:**

C_34_H_16_O_5_	*Z* = 4
*M**_r_* = 504.47	*F*(000) = 1040
Triclinic, *P*1	*D*_x_ = 1.406 Mg m^−^^3^
Hall symbol: -P 1	Mo *K*α radiation, λ = 0.71073 Å
*a* = 12.230 (4) Å	Cell parameters from 182 reflections
*b* = 12.346 (3) Å	θ = 3.3–21.5°
*c* = 16.794 (5) Å	µ = 0.10 mm^−^^1^
α = 82.229 (7)°	*T* = 296 K
β = 72.773 (6)°	Prism, dark green
γ = 81.181 (10)°	0.28 × 0.18 × 0.14 mm
*V* = 2382.5 (12) Å^3^	

### Data collection

**Table:**

Bruker Kappa APEXII CCD diffractometer	10895 independent reflections
Radiation source: fine-focus sealed tube	4858 reflections with *I* > 2σ(*I*)
Graphite monochromator	*R*_int_ = 0.061
Detector resolution: 0.81 pixels mm^-1^	θ_max_ = 27.6°, θ_min_ = 2.3°
ω scans	*h* = −15→15
Absorption correction: multi-scan (*SADABS*; Bruker, 2005)	*k* = −16→16
*T*_min_ = 0.980, *T*_max_ = 0.987	*l* = −21→21
38137 measured reflections	

### Refinement

**Table:**

Refinement on *F*^2^	Secondary atom site location: difference Fourier map
Least-squares matrix: full	Hydrogen site location: inferred from neighbouring sites
*R*[*F*^2^ > 2σ(*F*^2^)] = 0.056	H-atom parameters constrained
*wR*(*F*^2^) = 0.135	*w* = 1/[σ^2^(*F*_o_^2^) + (0.0501*P*)^2^] where *P* = (*F*_o_^2^ + 2*F*_c_^2^)/3
*S* = 0.94	(Δ/σ)_max_ < 0.001
10895 reflections	Δρ_max_ = 0.19 e Å^−^^3^
704 parameters	Δρ_min_ = −0.21 e Å^−^^3^
0 restraints	Extinction correction: *SHELXL97* (Sheldrick, 2008), FC^*^=KFC[1+0.001XFC^2^Λ^3^/SIN(2Θ)]^-1/4^
Primary atom site location: structure-invariant direct methods	Extinction coefficient: 0.0020 (4)

### Special details

**Table:**

Geometry. Bond distances, angles *etc*. have been calculated using the rounded fractional coordinates. All su's are estimated from the variances of the (full) variance-covariance matrix. The cell e.s.d.'s are taken into account in the estimation of distances, angles and torsion angles
Refinement. Refinement on *F*^2^ for ALL reflections except those flagged by the user for potential systematic errors. Weighted *R*-factors *wR* and all goodnesses of fit *S* are based on *F*^2^, conventional *R*-factors *R* are based on *F*, with *F* set to zero for negative *F*^2^. The observed criterion of *F*^2^ > σ(*F*^2^) is used only for calculating -*R*-factor-obs *etc*. and is not relevant to the choice of reflections for refinement. *R*-factors based on *F*^2^ are statistically about twice as large as those based on *F*, and *R*-factors based on ALL data will be even larger.

### Fractional atomic coordinates and isotropic or equivalent isotropic displacement parameters (Å^2^)

**Table:**

	*x*	*y*	*z*	*U*_iso_*/*U*_eq_	
O1	0.30343 (13)	0.59668 (14)	0.68545 (12)	0.0606 (7)	
O2	0.36189 (17)	0.52360 (19)	0.56612 (14)	0.0919 (10)	
O3	0.65201 (14)	0.36139 (15)	0.71664 (13)	0.0755 (8)	
O4	0.31239 (15)	0.02705 (15)	0.72691 (12)	0.0721 (8)	
O5	0.12537 (14)	0.48837 (16)	0.91275 (12)	0.0710 (8)	
C1	0.36124 (18)	0.5327 (2)	0.80850 (17)	0.0457 (9)	
C2	0.3815 (2)	0.5551 (3)	0.88099 (18)	0.0660 (11)	
C3	0.3712 (2)	0.6644 (3)	0.8990 (2)	0.0826 (16)	
C4	0.3414 (2)	0.7490 (3)	0.8436 (3)	0.0847 (16)	
C5	0.3211 (2)	0.7288 (2)	0.7722 (2)	0.0709 (13)	
C6	0.33166 (19)	0.6210 (2)	0.75593 (17)	0.0495 (10)	
C7	0.3894 (2)	0.5616 (2)	0.61735 (19)	0.0570 (11)	
C8	0.5070 (2)	0.5881 (2)	0.61130 (15)	0.0474 (9)	
C9	0.5342 (3)	0.6868 (2)	0.56465 (18)	0.0692 (12)	
C10	0.6393 (3)	0.7211 (3)	0.5530 (2)	0.0821 (14)	
C11	0.7199 (3)	0.6572 (3)	0.5856 (2)	0.0797 (15)	
C12	0.6954 (2)	0.5587 (2)	0.63184 (18)	0.0616 (11)	
C13	0.58751 (19)	0.5234 (2)	0.64604 (15)	0.0431 (9)	
C14	0.57028 (19)	0.4167 (2)	0.69716 (16)	0.0462 (9)	
C15	0.45753 (17)	0.36805 (19)	0.72787 (14)	0.0400 (9)	
C16	0.36222 (19)	0.41890 (19)	0.78720 (15)	0.0422 (9)	
C17	0.26743 (18)	0.3624 (2)	0.82399 (14)	0.0415 (9)	
C18	0.26223 (18)	0.2556 (2)	0.80507 (14)	0.0407 (8)	
C19	0.35423 (18)	0.20753 (19)	0.74519 (14)	0.0403 (8)	
C20	0.45172 (18)	0.2643 (2)	0.70666 (15)	0.0416 (9)	
C21	0.53516 (18)	0.1954 (2)	0.64342 (15)	0.0445 (9)	
C22	0.6417 (2)	0.2118 (2)	0.58698 (17)	0.0609 (10)	
C23	0.6949 (2)	0.1317 (2)	0.53227 (18)	0.0672 (11)	
C24	0.6467 (2)	0.0389 (2)	0.53273 (18)	0.0659 (11)	
C25	0.5414 (2)	0.0214 (2)	0.58938 (17)	0.0585 (10)	
C26	0.48748 (19)	0.1004 (2)	0.64358 (15)	0.0449 (9)	
C27	0.3741 (2)	0.0999 (2)	0.70820 (16)	0.0482 (10)	
C28	0.1557 (2)	0.3987 (2)	0.88562 (16)	0.0477 (9)	
C29	0.08788 (19)	0.3041 (2)	0.90507 (15)	0.0456 (9)	
C30	−0.02000 (19)	0.2945 (2)	0.95961 (16)	0.0532 (10)	
C31	−0.0662 (2)	0.1982 (2)	0.96602 (17)	0.0588 (10)	
C32	−0.0065 (2)	0.1132 (2)	0.91909 (16)	0.0556 (10)	
C33	0.10298 (19)	0.1220 (2)	0.86364 (15)	0.0503 (9)	
C34	0.15016 (18)	0.2189 (2)	0.85727 (15)	0.0417 (9)	
O6	1.00908 (13)	−0.17362 (14)	0.77863 (11)	0.0535 (7)	
O7	0.90062 (15)	−0.12612 (16)	0.90231 (12)	0.0674 (8)	
O8	0.63534 (16)	−0.13362 (14)	0.72125 (13)	0.0714 (8)	
O9	0.68213 (15)	0.30354 (15)	0.88768 (11)	0.0639 (7)	
O10	1.11392 (14)	0.04935 (15)	0.60688 (12)	0.0679 (7)	
C35	0.94112 (18)	−0.11759 (19)	0.65889 (16)	0.0419 (9)	
C36	0.9417 (2)	−0.1352 (2)	0.57890 (17)	0.0529 (10)	
C37	1.0053 (2)	−0.2271 (2)	0.54203 (17)	0.0577 (10)	
C38	1.0671 (2)	−0.3024 (2)	0.5844 (2)	0.0609 (10)	
C39	1.06743 (19)	−0.2873 (2)	0.66449 (19)	0.0557 (10)	
C40	1.00473 (18)	−0.1955 (2)	0.69976 (16)	0.0433 (9)	
C41	0.9114 (2)	−0.1772 (2)	0.84396 (18)	0.0483 (10)	
C42	0.83080 (18)	−0.25756 (19)	0.84404 (15)	0.0409 (8)	
C43	0.8502 (2)	−0.3585 (2)	0.88940 (15)	0.0504 (10)	
C44	0.7807 (2)	−0.4394 (2)	0.89913 (17)	0.0586 (11)	
C45	0.6915 (2)	−0.4225 (2)	0.86307 (17)	0.0573 (10)	
C46	0.67236 (19)	−0.3245 (2)	0.81736 (16)	0.0498 (10)	
C47	0.74017 (18)	−0.23984 (19)	0.80699 (14)	0.0389 (8)	
C48	0.70772 (19)	−0.1365 (2)	0.75803 (16)	0.0453 (9)	
C49	0.76039 (18)	−0.03298 (19)	0.75539 (15)	0.0407 (8)	
C50	0.87355 (18)	−0.02196 (19)	0.70404 (14)	0.0408 (8)	
C51	0.91980 (18)	0.0738 (2)	0.70171 (14)	0.0411 (8)	
C52	0.85951 (18)	0.16119 (19)	0.74945 (14)	0.0391 (8)	
C53	0.74977 (18)	0.14884 (18)	0.80130 (14)	0.0384 (8)	
C54	0.69851 (18)	0.05350 (19)	0.80181 (14)	0.0383 (8)	
C55	0.57903 (18)	0.0654 (2)	0.85817 (15)	0.0416 (8)	
C56	0.49093 (19)	−0.0013 (2)	0.87878 (16)	0.0531 (10)	
C57	0.3861 (2)	0.0352 (2)	0.93379 (17)	0.0606 (11)	
C58	0.3681 (2)	0.1337 (3)	0.96666 (17)	0.0616 (10)	
C59	0.4552 (2)	0.2002 (2)	0.94781 (15)	0.0542 (10)	
C60	0.55958 (19)	0.1651 (2)	0.89297 (15)	0.0433 (9)	
C61	0.66597 (19)	0.2206 (2)	0.86300 (15)	0.0443 (9)	
C62	1.0373 (2)	0.1048 (2)	0.65342 (16)	0.0490 (10)	
C63	1.03846 (19)	0.2175 (2)	0.67471 (15)	0.0456 (9)	
C64	1.1243 (2)	0.2854 (2)	0.64486 (16)	0.0558 (10)	
C65	1.1039 (2)	0.3894 (3)	0.67096 (18)	0.0656 (11)	
C66	0.9996 (2)	0.4235 (2)	0.72629 (18)	0.0645 (11)	
C67	0.9133 (2)	0.3553 (2)	0.75702 (16)	0.0538 (10)	
C68	0.93334 (19)	0.2515 (2)	0.73056 (15)	0.0418 (9)	
H2	0.40190	0.49770	0.91780	0.0790*	
H3	0.38440	0.68000	0.94780	0.0990*	
H4	0.33520	0.82140	0.85550	0.1020*	
H5	0.30060	0.78610	0.73540	0.0850*	
H9	0.48030	0.73010	0.54110	0.0830*	
H10	0.65600	0.78820	0.52260	0.0980*	
H11	0.79180	0.68040	0.57660	0.0960*	
H12	0.75090	0.51540	0.65370	0.0740*	
H22	0.67620	0.27430	0.58580	0.0730*	
H23	0.76600	0.14180	0.49400	0.0810*	
H24	0.68460	−0.01250	0.49500	0.0790*	
H25	0.50800	−0.04180	0.59090	0.0700*	
H30	−0.06040	0.35170	0.99120	0.0640*	
H31	−0.13900	0.19010	1.00250	0.0710*	
H32	−0.03970	0.04870	0.92450	0.0670*	
H33	0.14290	0.06460	0.83200	0.0600*	
H36	0.89900	−0.08480	0.54990	0.0630*	
H37	1.00590	−0.23780	0.48810	0.0690*	
H38	1.10920	−0.36430	0.55920	0.0730*	
H39	1.10930	−0.33840	0.69360	0.0670*	
H43	0.91110	−0.37100	0.91330	0.0610*	
H44	0.79390	−0.50580	0.93020	0.0700*	
H45	0.64430	−0.47730	0.86970	0.0690*	
H46	0.61240	−0.31420	0.79250	0.0600*	
H56	0.50180	−0.06840	0.85640	0.0640*	
H57	0.32650	−0.00880	0.94860	0.0730*	
H58	0.29630	0.15630	1.00220	0.0740*	
H59	0.44400	0.26650	0.97130	0.0650*	
H64	1.19450	0.26140	0.60790	0.0670*	
H65	1.16040	0.43670	0.65130	0.0790*	
H66	0.98690	0.49400	0.74340	0.0770*	
H67	0.84370	0.37910	0.79460	0.0650*	

### Atomic displacement parameters (Å^2^)

**Table:**

	*U*^11^	*U*^22^	*U*^33^	*U*^12^	*U*^13^	*U*^23^
O1	0.0502 (10)	0.0595 (13)	0.0732 (14)	0.0036 (9)	−0.0241 (10)	−0.0066 (11)
O2	0.0874 (15)	0.120 (2)	0.0876 (17)	−0.0121 (13)	−0.0433 (13)	−0.0343 (15)
O3	0.0540 (11)	0.0714 (14)	0.1047 (17)	−0.0081 (10)	−0.0386 (11)	0.0168 (12)
O4	0.0663 (12)	0.0524 (13)	0.0930 (16)	−0.0217 (10)	−0.0010 (11)	−0.0229 (11)
O5	0.0637 (11)	0.0605 (14)	0.0801 (15)	−0.0056 (10)	0.0023 (10)	−0.0303 (12)
C1	0.0398 (13)	0.0489 (18)	0.0484 (17)	−0.0110 (12)	−0.0061 (12)	−0.0121 (15)
C2	0.0640 (17)	0.079 (2)	0.058 (2)	−0.0239 (16)	−0.0094 (15)	−0.0167 (18)
C3	0.074 (2)	0.108 (3)	0.071 (3)	−0.039 (2)	0.0024 (18)	−0.046 (2)
C4	0.066 (2)	0.070 (3)	0.113 (3)	−0.0272 (18)	0.009 (2)	−0.047 (3)
C5	0.0593 (18)	0.045 (2)	0.103 (3)	−0.0059 (14)	−0.0089 (18)	−0.0208 (19)
C6	0.0387 (13)	0.0469 (19)	0.060 (2)	−0.0056 (12)	−0.0047 (13)	−0.0153 (16)
C7	0.0576 (17)	0.0535 (19)	0.060 (2)	0.0011 (14)	−0.0209 (16)	−0.0051 (16)
C8	0.0522 (15)	0.0397 (17)	0.0469 (17)	−0.0037 (13)	−0.0083 (13)	−0.0073 (14)
C9	0.078 (2)	0.052 (2)	0.065 (2)	−0.0009 (16)	−0.0078 (17)	0.0024 (17)
C10	0.092 (2)	0.048 (2)	0.083 (3)	−0.0186 (19)	0.014 (2)	−0.0023 (18)
C11	0.0650 (19)	0.076 (3)	0.090 (3)	−0.0323 (18)	0.0043 (18)	−0.012 (2)
C12	0.0518 (16)	0.063 (2)	0.069 (2)	−0.0189 (14)	−0.0096 (14)	−0.0059 (17)
C13	0.0436 (13)	0.0426 (16)	0.0430 (16)	−0.0102 (12)	−0.0084 (12)	−0.0071 (13)
C14	0.0397 (13)	0.0490 (17)	0.0516 (17)	−0.0043 (12)	−0.0153 (12)	−0.0067 (14)
C15	0.0401 (13)	0.0363 (16)	0.0442 (16)	−0.0060 (11)	−0.0122 (12)	−0.0037 (13)
C16	0.0449 (13)	0.0376 (16)	0.0455 (16)	−0.0076 (11)	−0.0138 (12)	−0.0031 (13)
C17	0.0419 (13)	0.0399 (16)	0.0440 (16)	−0.0050 (11)	−0.0125 (12)	−0.0079 (13)
C18	0.0410 (13)	0.0414 (16)	0.0409 (15)	−0.0057 (11)	−0.0132 (12)	−0.0031 (13)
C19	0.0428 (13)	0.0373 (16)	0.0404 (15)	−0.0049 (11)	−0.0105 (12)	−0.0054 (12)
C20	0.0425 (13)	0.0378 (16)	0.0435 (16)	−0.0050 (11)	−0.0124 (12)	0.0006 (13)
C21	0.0409 (13)	0.0420 (17)	0.0479 (17)	−0.0008 (12)	−0.0112 (12)	−0.0022 (13)
C22	0.0512 (15)	0.0572 (19)	0.066 (2)	−0.0088 (14)	−0.0021 (15)	−0.0073 (16)
C23	0.0484 (15)	0.066 (2)	0.071 (2)	−0.0019 (15)	0.0081 (15)	−0.0130 (18)
C24	0.0563 (17)	0.062 (2)	0.065 (2)	0.0077 (15)	0.0011 (15)	−0.0147 (17)
C25	0.0583 (16)	0.0475 (18)	0.067 (2)	0.0006 (13)	−0.0134 (15)	−0.0134 (16)
C26	0.0454 (13)	0.0424 (17)	0.0434 (16)	0.0007 (12)	−0.0094 (12)	−0.0061 (13)
C27	0.0484 (14)	0.0433 (18)	0.0525 (18)	−0.0041 (13)	−0.0125 (13)	−0.0087 (14)
C28	0.0477 (14)	0.0460 (18)	0.0477 (17)	−0.0008 (13)	−0.0096 (12)	−0.0126 (14)
C29	0.0430 (13)	0.0502 (18)	0.0448 (16)	−0.0072 (12)	−0.0126 (12)	−0.0059 (14)
C30	0.0469 (15)	0.062 (2)	0.0496 (18)	−0.0067 (13)	−0.0094 (13)	−0.0110 (15)
C31	0.0500 (15)	0.071 (2)	0.0520 (19)	−0.0132 (15)	−0.0069 (13)	−0.0040 (16)
C32	0.0579 (16)	0.059 (2)	0.0522 (18)	−0.0233 (14)	−0.0149 (14)	0.0039 (15)
C33	0.0492 (14)	0.0487 (18)	0.0512 (17)	−0.0084 (13)	−0.0101 (13)	−0.0046 (14)
C34	0.0432 (13)	0.0412 (16)	0.0413 (16)	−0.0084 (12)	−0.0123 (12)	−0.0014 (13)
O6	0.0445 (10)	0.0616 (13)	0.0568 (12)	−0.0101 (8)	−0.0177 (9)	−0.0017 (10)
O7	0.0801 (13)	0.0696 (14)	0.0628 (14)	−0.0236 (10)	−0.0230 (11)	−0.0188 (11)
O8	0.0838 (13)	0.0526 (13)	0.0974 (16)	−0.0077 (10)	−0.0588 (12)	−0.0003 (11)
O9	0.0766 (12)	0.0522 (13)	0.0598 (13)	−0.0122 (10)	−0.0051 (10)	−0.0212 (10)
O10	0.0512 (10)	0.0734 (14)	0.0675 (14)	−0.0011 (10)	0.0048 (10)	−0.0224 (11)
C35	0.0390 (12)	0.0360 (16)	0.0491 (17)	−0.0022 (11)	−0.0097 (12)	−0.0078 (13)
C36	0.0554 (15)	0.0476 (18)	0.0556 (19)	−0.0014 (13)	−0.0173 (14)	−0.0066 (15)
C37	0.0607 (16)	0.056 (2)	0.0534 (18)	−0.0045 (14)	−0.0071 (14)	−0.0182 (16)
C38	0.0513 (16)	0.0441 (18)	0.076 (2)	0.0000 (13)	0.0015 (15)	−0.0173 (17)
C39	0.0391 (13)	0.0434 (18)	0.078 (2)	0.0013 (12)	−0.0110 (14)	−0.0025 (16)
C40	0.0349 (12)	0.0421 (17)	0.0497 (17)	−0.0046 (11)	−0.0065 (12)	−0.0061 (14)
C41	0.0534 (16)	0.0418 (17)	0.0538 (19)	−0.0029 (13)	−0.0230 (15)	−0.0035 (15)
C42	0.0463 (13)	0.0345 (15)	0.0429 (16)	−0.0040 (11)	−0.0125 (12)	−0.0085 (13)
C43	0.0541 (15)	0.0461 (18)	0.0496 (17)	0.0006 (13)	−0.0155 (13)	−0.0048 (14)
C44	0.0695 (18)	0.0395 (18)	0.062 (2)	−0.0041 (14)	−0.0153 (15)	0.0013 (15)
C45	0.0615 (16)	0.0373 (18)	0.073 (2)	−0.0146 (13)	−0.0149 (15)	−0.0038 (15)
C46	0.0473 (14)	0.0411 (17)	0.0625 (19)	−0.0057 (12)	−0.0148 (13)	−0.0107 (14)
C47	0.0434 (13)	0.0293 (14)	0.0438 (15)	−0.0015 (11)	−0.0114 (11)	−0.0085 (12)
C48	0.0449 (14)	0.0388 (17)	0.0537 (17)	−0.0023 (12)	−0.0152 (13)	−0.0092 (13)
C49	0.0447 (13)	0.0330 (15)	0.0452 (16)	−0.0010 (11)	−0.0173 (12)	0.0001 (12)
C50	0.0447 (13)	0.0365 (16)	0.0402 (15)	0.0008 (12)	−0.0126 (12)	−0.0047 (12)
C51	0.0396 (13)	0.0398 (16)	0.0417 (15)	−0.0017 (12)	−0.0107 (11)	−0.0012 (13)
C52	0.0446 (13)	0.0345 (15)	0.0378 (15)	−0.0021 (11)	−0.0132 (12)	−0.0017 (12)
C53	0.0441 (13)	0.0302 (15)	0.0385 (15)	−0.0020 (11)	−0.0099 (11)	−0.0018 (12)
C54	0.0410 (12)	0.0326 (15)	0.0391 (15)	−0.0015 (11)	−0.0110 (11)	0.0005 (12)
C55	0.0433 (13)	0.0377 (16)	0.0411 (15)	−0.0033 (12)	−0.0116 (11)	0.0031 (13)
C56	0.0494 (15)	0.0491 (18)	0.0607 (19)	−0.0084 (13)	−0.0141 (14)	−0.0049 (14)
C57	0.0477 (16)	0.069 (2)	0.060 (2)	−0.0134 (14)	−0.0068 (14)	0.0000 (17)
C58	0.0437 (15)	0.081 (2)	0.0511 (19)	0.0006 (15)	−0.0039 (13)	−0.0053 (17)
C59	0.0556 (16)	0.0553 (19)	0.0451 (17)	0.0032 (14)	−0.0065 (13)	−0.0103 (14)
C60	0.0468 (14)	0.0416 (17)	0.0359 (15)	−0.0014 (12)	−0.0077 (12)	0.0020 (13)
C61	0.0544 (15)	0.0363 (16)	0.0381 (16)	0.0004 (12)	−0.0098 (12)	−0.0035 (13)
C62	0.0439 (14)	0.0572 (19)	0.0432 (17)	−0.0035 (13)	−0.0097 (13)	−0.0041 (14)
C63	0.0490 (15)	0.0465 (17)	0.0412 (16)	−0.0106 (13)	−0.0118 (12)	−0.0005 (13)
C64	0.0530 (15)	0.067 (2)	0.0444 (17)	−0.0185 (15)	−0.0061 (13)	0.0002 (15)
C65	0.0689 (19)	0.063 (2)	0.067 (2)	−0.0283 (16)	−0.0184 (17)	0.0078 (17)
C66	0.079 (2)	0.0478 (19)	0.072 (2)	−0.0239 (16)	−0.0221 (18)	−0.0035 (16)
C67	0.0564 (15)	0.0445 (18)	0.0618 (19)	−0.0145 (13)	−0.0151 (14)	−0.0030 (15)
C68	0.0473 (14)	0.0402 (16)	0.0406 (15)	−0.0107 (12)	−0.0151 (12)	−0.0003 (13)

### Geometric parameters (Å, º)

**Table:**

O1—C6	1.407 (3)	C24—H24	0.9300
O1—C7	1.372 (3)	C25—H25	0.9300
O2—C7	1.184 (4)	C30—H30	0.9300
O3—C14	1.223 (3)	C31—H31	0.9300
O4—C27	1.212 (3)	C32—H32	0.9300
O5—C28	1.217 (3)	C33—H33	0.9300
O6—C40	1.405 (3)	C35—C36	1.387 (4)
O6—C41	1.364 (3)	C35—C40	1.382 (3)
O7—C41	1.200 (3)	C35—C50	1.491 (3)
O8—C48	1.214 (3)	C36—C37	1.382 (4)
O9—C61	1.219 (3)	C37—C38	1.366 (4)
O10—C62	1.213 (3)	C38—C39	1.384 (4)
C1—C16	1.493 (3)	C39—C40	1.367 (4)
C1—C6	1.381 (4)	C41—C42	1.502 (4)
C1—C2	1.382 (4)	C42—C43	1.396 (3)
C2—C3	1.401 (5)	C42—C47	1.400 (3)
C3—C4	1.382 (5)	C43—C44	1.371 (4)
C4—C5	1.356 (5)	C44—C45	1.374 (4)
C5—C6	1.374 (4)	C45—C46	1.370 (4)
C7—C8	1.495 (4)	C46—C47	1.393 (3)
C8—C13	1.386 (4)	C47—C48	1.489 (3)
C8—C9	1.386 (4)	C48—C49	1.507 (3)
C9—C10	1.366 (5)	C49—C50	1.413 (3)
C10—C11	1.363 (5)	C49—C54	1.396 (3)
C11—C12	1.375 (4)	C50—C51	1.378 (3)
C12—C13	1.396 (4)	C51—C52	1.411 (3)
C13—C14	1.480 (4)	C51—C62	1.505 (4)
C14—C15	1.509 (3)	C52—C53	1.385 (3)
C15—C20	1.392 (3)	C52—C68	1.484 (3)
C15—C16	1.415 (3)	C53—C54	1.414 (3)
C16—C17	1.385 (3)	C53—C61	1.500 (3)
C17—C28	1.496 (3)	C54—C55	1.485 (3)
C17—C18	1.412 (3)	C55—C56	1.390 (3)
C18—C19	1.384 (3)	C55—C60	1.391 (3)
C18—C34	1.491 (3)	C56—C57	1.391 (4)
C19—C27	1.499 (3)	C57—C58	1.364 (4)
C19—C20	1.417 (3)	C58—C59	1.381 (4)
C20—C21	1.493 (3)	C59—C60	1.381 (4)
C21—C22	1.391 (4)	C60—C61	1.486 (4)
C21—C26	1.387 (3)	C62—C63	1.486 (4)
C22—C23	1.393 (4)	C63—C64	1.379 (4)
C23—C24	1.365 (4)	C63—C68	1.391 (3)
C24—C25	1.384 (4)	C64—C65	1.376 (4)
C25—C26	1.379 (4)	C65—C66	1.381 (4)
C26—C27	1.487 (4)	C66—C67	1.386 (4)
C28—C29	1.481 (4)	C67—C68	1.376 (4)
C29—C30	1.377 (4)	C36—H36	0.9300
C29—C34	1.397 (3)	C37—H37	0.9300
C30—C31	1.371 (4)	C38—H38	0.9300
C31—C32	1.380 (4)	C39—H39	0.9300
C32—C33	1.397 (4)	C43—H43	0.9300
C33—C34	1.384 (3)	C44—H44	0.9300
C2—H2	0.9300	C45—H45	0.9300
C3—H3	0.9300	C46—H46	0.9300
C4—H4	0.9300	C56—H56	0.9300
C5—H5	0.9300	C57—H57	0.9300
C9—H9	0.9300	C58—H58	0.9300
C10—H10	0.9300	C59—H59	0.9300
C11—H11	0.9300	C64—H64	0.9300
C12—H12	0.9300	C65—H65	0.9300
C22—H22	0.9300	C66—H66	0.9300
C23—H23	0.9300	C67—H67	0.9300
			
C6—O1—C7	119.8 (2)	C34—C33—H33	121.00
C40—O6—C41	118.32 (19)	C36—C35—C40	117.6 (2)
C2—C1—C6	117.7 (3)	C36—C35—C50	124.1 (2)
C6—C1—C16	118.7 (2)	C40—C35—C50	118.3 (2)
C2—C1—C16	123.5 (3)	C35—C36—C37	120.4 (2)
C1—C2—C3	119.9 (3)	C36—C37—C38	120.3 (3)
C2—C3—C4	119.5 (3)	C37—C38—C39	120.5 (2)
C3—C4—C5	121.5 (3)	C38—C39—C40	118.4 (2)
C4—C5—C6	117.9 (3)	O6—C40—C35	117.1 (2)
O1—C6—C1	116.9 (2)	O6—C40—C39	120.0 (2)
C1—C6—C5	123.5 (3)	C35—C40—C39	122.8 (2)
O1—C6—C5	119.4 (2)	O6—C41—O7	118.8 (2)
O1—C7—C8	115.4 (2)	O6—C41—C42	117.4 (2)
O2—C7—C8	126.7 (3)	O7—C41—C42	123.2 (2)
O1—C7—O2	117.5 (2)	C41—C42—C43	113.4 (2)
C7—C8—C9	114.1 (3)	C41—C42—C47	127.3 (2)
C9—C8—C13	119.6 (3)	C43—C42—C47	119.3 (2)
C7—C8—C13	126.2 (2)	C42—C43—C44	120.9 (2)
C8—C9—C10	120.6 (3)	C43—C44—C45	120.1 (2)
C9—C10—C11	120.3 (3)	C44—C45—C46	119.7 (2)
C10—C11—C12	120.4 (3)	C45—C46—C47	121.8 (2)
C11—C12—C13	120.3 (3)	C42—C47—C46	118.2 (2)
C8—C13—C12	118.9 (2)	C42—C47—C48	125.6 (2)
C12—C13—C14	115.6 (2)	C46—C47—C48	116.3 (2)
C8—C13—C14	125.5 (2)	O8—C48—C47	120.1 (2)
O3—C14—C13	119.3 (2)	O8—C48—C49	118.8 (2)
C13—C14—C15	124.9 (2)	C47—C48—C49	121.1 (2)
O3—C14—C15	115.8 (2)	C48—C49—C50	119.7 (2)
C14—C15—C20	119.5 (2)	C48—C49—C54	121.1 (2)
C16—C15—C20	119.0 (2)	C50—C49—C54	119.2 (2)
C14—C15—C16	120.9 (2)	C35—C50—C49	118.5 (2)
C1—C16—C17	120.0 (2)	C35—C50—C51	122.5 (2)
C15—C16—C17	118.8 (2)	C49—C50—C51	118.9 (2)
C1—C16—C15	121.1 (2)	C50—C51—C52	122.8 (2)
C16—C17—C28	129.3 (2)	C50—C51—C62	129.1 (2)
C18—C17—C28	108.1 (2)	C52—C51—C62	108.1 (2)
C16—C17—C18	122.7 (2)	C51—C52—C53	118.1 (2)
C17—C18—C19	118.3 (2)	C51—C52—C68	108.5 (2)
C19—C18—C34	133.3 (2)	C53—C52—C68	133.4 (2)
C17—C18—C34	108.4 (2)	C52—C53—C54	120.1 (2)
C18—C19—C20	119.8 (2)	C52—C53—C61	132.1 (2)
C20—C19—C27	108.3 (2)	C54—C53—C61	107.9 (2)
C18—C19—C27	132.0 (2)	C49—C54—C53	120.8 (2)
C15—C20—C21	130.3 (2)	C49—C54—C55	130.5 (2)
C19—C20—C21	108.3 (2)	C53—C54—C55	108.6 (2)
C15—C20—C19	121.4 (2)	C54—C55—C56	132.4 (2)
C20—C21—C22	132.7 (2)	C54—C55—C60	108.3 (2)
C20—C21—C26	108.1 (2)	C56—C55—C60	119.3 (2)
C22—C21—C26	119.1 (2)	C55—C56—C57	118.1 (2)
C21—C22—C23	117.9 (2)	C56—C57—C58	121.9 (2)
C22—C23—C24	122.5 (3)	C57—C58—C59	120.8 (3)
C23—C24—C25	120.0 (2)	C58—C59—C60	117.9 (2)
C24—C25—C26	118.1 (2)	C55—C60—C59	122.1 (2)
C21—C26—C25	122.5 (2)	C55—C60—C61	109.5 (2)
C21—C26—C27	110.1 (2)	C59—C60—C61	128.4 (2)
C25—C26—C27	127.4 (2)	O9—C61—C53	128.1 (2)
O4—C27—C19	128.0 (2)	O9—C61—C60	126.4 (2)
O4—C27—C26	126.9 (2)	C53—C61—C60	105.4 (2)
C19—C27—C26	105.1 (2)	O10—C62—C51	127.0 (2)
O5—C28—C29	126.9 (2)	O10—C62—C63	127.8 (2)
C17—C28—C29	105.9 (2)	C51—C62—C63	105.2 (2)
O5—C28—C17	127.2 (2)	C62—C63—C64	128.8 (2)
C28—C29—C30	128.9 (2)	C62—C63—C68	109.6 (2)
C30—C29—C34	121.8 (2)	C64—C63—C68	121.6 (2)
C28—C29—C34	109.3 (2)	C63—C64—C65	118.5 (2)
C29—C30—C31	118.2 (2)	C64—C65—C66	120.0 (3)
C30—C31—C32	121.0 (2)	C65—C66—C67	121.7 (3)
C31—C32—C33	121.3 (2)	C66—C67—C68	118.3 (2)
C32—C33—C34	117.9 (2)	C52—C68—C63	108.6 (2)
C18—C34—C29	108.3 (2)	C52—C68—C67	131.6 (2)
C29—C34—C33	119.8 (2)	C63—C68—C67	119.8 (2)
C18—C34—C33	131.8 (2)	C35—C36—H36	120.00
C1—C2—H2	120.00	C37—C36—H36	120.00
C3—C2—H2	120.00	C36—C37—H37	120.00
C4—C3—H3	120.00	C38—C37—H37	120.00
C2—C3—H3	120.00	C37—C38—H38	120.00
C5—C4—H4	119.00	C39—C38—H38	120.00
C3—C4—H4	119.00	C38—C39—H39	121.00
C4—C5—H5	121.00	C40—C39—H39	121.00
C6—C5—H5	121.00	C42—C43—H43	120.00
C8—C9—H9	120.00	C44—C43—H43	120.00
C10—C9—H9	120.00	C43—C44—H44	120.00
C11—C10—H10	120.00	C45—C44—H44	120.00
C9—C10—H10	120.00	C44—C45—H45	120.00
C10—C11—H11	120.00	C46—C45—H45	120.00
C12—C11—H11	120.00	C45—C46—H46	119.00
C13—C12—H12	120.00	C47—C46—H46	119.00
C11—C12—H12	120.00	C55—C56—H56	121.00
C21—C22—H22	121.00	C57—C56—H56	121.00
C23—C22—H22	121.00	C56—C57—H57	119.00
C24—C23—H23	119.00	C58—C57—H57	119.00
C22—C23—H23	119.00	C57—C58—H58	120.00
C23—C24—H24	120.00	C59—C58—H58	120.00
C25—C24—H24	120.00	C58—C59—H59	121.00
C26—C25—H25	121.00	C60—C59—H59	121.00
C24—C25—H25	121.00	C63—C64—H64	121.00
C29—C30—H30	121.00	C65—C64—H64	121.00
C31—C30—H30	121.00	C64—C65—H65	120.00
C32—C31—H31	119.00	C66—C65—H65	120.00
C30—C31—H31	119.00	C65—C66—H66	119.00
C31—C32—H32	119.00	C67—C66—H66	119.00
C33—C32—H32	119.00	C66—C67—H67	121.00
C32—C33—H33	121.00	C68—C67—H67	121.00
			
C7—O1—C6—C1	−76.7 (3)	C30—C31—C32—C33	0.2 (4)
C7—O1—C6—C5	107.8 (3)	C31—C32—C33—C34	0.2 (4)
C6—O1—C7—O2	166.9 (2)	C32—C33—C34—C18	−179.5 (2)
C6—O1—C7—C8	−20.1 (3)	C32—C33—C34—C29	−0.5 (4)
C40—O6—C41—C42	32.3 (3)	C50—C35—C40—O6	−5.5 (3)
C41—O6—C40—C35	69.9 (3)	C50—C35—C40—C39	178.5 (2)
C41—O6—C40—C39	−114.0 (3)	C36—C35—C50—C49	90.7 (3)
C40—O6—C41—O7	−156.0 (2)	C36—C35—C50—C51	−93.5 (3)
C16—C1—C6—C5	175.9 (2)	C40—C35—C50—C49	−87.4 (3)
C2—C1—C16—C15	−102.7 (3)	C40—C35—C50—C51	88.4 (3)
C2—C1—C16—C17	79.1 (3)	C36—C35—C40—O6	176.3 (2)
C16—C1—C6—O1	0.5 (3)	C36—C35—C40—C39	0.3 (4)
C16—C1—C2—C3	−175.6 (2)	C40—C35—C36—C37	−0.8 (4)
C2—C1—C6—O1	−175.7 (2)	C50—C35—C36—C37	−178.9 (2)
C2—C1—C6—C5	−0.3 (4)	C35—C36—C37—C38	0.9 (4)
C6—C1—C16—C15	81.4 (3)	C36—C37—C38—C39	−0.4 (4)
C6—C1—C16—C17	−96.8 (3)	C37—C38—C39—C40	−0.1 (4)
C6—C1—C2—C3	0.3 (4)	C38—C39—C40—O6	−175.8 (2)
C1—C2—C3—C4	−0.4 (4)	C38—C39—C40—C35	0.1 (4)
C2—C3—C4—C5	0.4 (5)	O6—C41—C42—C43	93.9 (3)
C3—C4—C5—C6	−0.4 (5)	O6—C41—C42—C47	−88.4 (3)
C4—C5—C6—C1	0.3 (4)	O7—C41—C42—C43	−77.4 (3)
C4—C5—C6—O1	175.6 (3)	O7—C41—C42—C47	100.3 (3)
O2—C7—C8—C13	−98.7 (4)	C41—C42—C43—C44	176.9 (2)
O1—C7—C8—C9	−92.2 (3)	C47—C42—C43—C44	−1.0 (4)
O1—C7—C8—C13	89.0 (3)	C41—C42—C47—C46	−177.4 (2)
O2—C7—C8—C9	80.1 (3)	C41—C42—C47—C48	1.4 (4)
C13—C8—C9—C10	−0.4 (4)	C43—C42—C47—C46	0.2 (3)
C7—C8—C13—C12	177.7 (2)	C43—C42—C47—C48	179.0 (2)
C7—C8—C9—C10	−179.3 (3)	C42—C43—C44—C45	0.9 (4)
C7—C8—C13—C14	−1.3 (4)	C43—C44—C45—C46	0.0 (4)
C9—C8—C13—C12	−1.1 (4)	C44—C45—C46—C47	−0.9 (4)
C9—C8—C13—C14	179.9 (2)	C45—C46—C47—C42	0.7 (4)
C8—C9—C10—C11	1.5 (5)	C45—C46—C47—C48	−178.2 (2)
C9—C10—C11—C12	−1.3 (5)	C42—C47—C48—O8	172.3 (2)
C10—C11—C12—C13	−0.2 (5)	C42—C47—C48—C49	−9.8 (4)
C11—C12—C13—C8	1.4 (4)	C46—C47—C48—O8	−8.8 (4)
C11—C12—C13—C14	−179.5 (3)	C46—C47—C48—C49	169.1 (2)
C12—C13—C14—C15	173.4 (2)	O8—C48—C49—C50	−104.7 (3)
C12—C13—C14—O3	−7.1 (4)	O8—C48—C49—C54	74.2 (3)
C8—C13—C14—O3	172.0 (2)	C47—C48—C49—C50	77.4 (3)
C8—C13—C14—C15	−7.5 (4)	C47—C48—C49—C54	−103.8 (3)
O3—C14—C15—C20	−57.0 (3)	C48—C49—C50—C35	−4.8 (3)
C13—C14—C15—C16	−66.4 (3)	C48—C49—C50—C51	179.3 (2)
C13—C14—C15—C20	122.5 (3)	C54—C49—C50—C35	176.3 (2)
O3—C14—C15—C16	114.1 (3)	C54—C49—C50—C51	0.4 (3)
C14—C15—C20—C19	169.2 (2)	C48—C49—C54—C53	177.8 (2)
C14—C15—C20—C21	−12.7 (4)	C48—C49—C54—C55	−0.7 (4)
C16—C15—C20—C19	−2.1 (3)	C50—C49—C54—C53	−3.3 (3)
C14—C15—C16—C1	12.4 (3)	C50—C49—C54—C55	178.1 (2)
C14—C15—C16—C17	−169.4 (2)	C35—C50—C51—C52	−174.9 (2)
C20—C15—C16—C1	−176.5 (2)	C35—C50—C51—C62	3.5 (4)
C20—C15—C16—C17	1.8 (3)	C49—C50—C51—C52	0.9 (4)
C16—C15—C20—C21	176.0 (2)	C49—C50—C51—C62	179.3 (2)
C15—C16—C17—C28	−177.8 (2)	C50—C51—C52—C53	0.7 (3)
C1—C16—C17—C18	178.6 (2)	C50—C51—C52—C68	−178.8 (2)
C1—C16—C17—C28	0.4 (4)	C62—C51—C52—C53	−177.9 (2)
C15—C16—C17—C18	0.4 (4)	C62—C51—C52—C68	2.5 (3)
C16—C17—C18—C34	178.5 (2)	C50—C51—C62—O10	0.0 (4)
C28—C17—C18—C19	176.3 (2)	C50—C51—C62—C63	179.6 (2)
C16—C17—C18—C19	−2.3 (4)	C52—C51—C62—O10	178.5 (3)
C18—C17—C28—C29	3.2 (3)	C52—C51—C62—C63	−1.8 (3)
C16—C17—C28—O5	3.2 (4)	C51—C52—C53—C54	−3.6 (3)
C16—C17—C28—C29	−178.4 (2)	C51—C52—C53—C61	176.0 (2)
C28—C17—C18—C34	−3.0 (3)	C68—C52—C53—C54	175.8 (2)
C18—C17—C28—O5	−175.2 (3)	C68—C52—C53—C61	−4.6 (5)
C17—C18—C19—C20	1.9 (3)	C51—C52—C68—C63	−2.3 (3)
C17—C18—C19—C27	−177.0 (2)	C51—C52—C68—C67	176.5 (3)
C19—C18—C34—C29	−177.5 (3)	C53—C52—C68—C63	178.3 (3)
C19—C18—C34—C33	1.7 (5)	C53—C52—C68—C67	−3.0 (5)
C17—C18—C34—C33	−179.3 (3)	C52—C53—C54—C49	5.0 (3)
C34—C18—C19—C20	−179.1 (2)	C52—C53—C54—C55	−176.2 (2)
C34—C18—C19—C27	2.1 (5)	C61—C53—C54—C49	−174.7 (2)
C17—C18—C34—C29	1.6 (3)	C61—C53—C54—C55	4.2 (3)
C18—C19—C20—C21	−178.3 (2)	C52—C53—C61—O9	−8.4 (4)
C27—C19—C20—C15	179.3 (2)	C52—C53—C61—C60	175.4 (2)
C27—C19—C20—C21	0.9 (3)	C54—C53—C61—O9	171.2 (2)
C18—C19—C20—C15	0.2 (4)	C54—C53—C61—C60	−5.1 (3)
C18—C19—C27—O4	−3.8 (5)	C49—C54—C55—C56	−3.9 (4)
C18—C19—C27—C26	176.9 (2)	C49—C54—C55—C60	177.1 (2)
C20—C19—C27—O4	177.3 (3)	C53—C54—C55—C56	177.5 (3)
C20—C19—C27—C26	−2.1 (3)	C53—C54—C55—C60	−1.6 (3)
C15—C20—C21—C26	−177.5 (2)	C54—C55—C56—C57	−178.9 (2)
C19—C20—C21—C22	178.0 (3)	C60—C55—C56—C57	0.1 (4)
C15—C20—C21—C22	−0.3 (5)	C54—C55—C60—C59	179.4 (2)
C19—C20—C21—C26	0.8 (3)	C54—C55—C60—C61	−1.7 (3)
C20—C21—C26—C27	−2.2 (3)	C56—C55—C60—C59	0.2 (4)
C22—C21—C26—C27	−179.8 (2)	C56—C55—C60—C61	179.1 (2)
C26—C21—C22—C23	0.8 (4)	C55—C56—C57—C58	0.6 (4)
C22—C21—C26—C25	−0.4 (4)	C56—C57—C58—C59	−1.6 (4)
C20—C21—C22—C23	−176.2 (3)	C57—C58—C59—C60	1.8 (4)
C20—C21—C26—C25	177.3 (2)	C58—C59—C60—C55	−1.1 (4)
C21—C22—C23—C24	−0.4 (4)	C58—C59—C60—C61	−179.8 (2)
C22—C23—C24—C25	−0.4 (4)	C55—C60—C61—O9	−172.2 (2)
C23—C24—C25—C26	0.8 (4)	C55—C60—C61—C53	4.1 (3)
C24—C25—C26—C21	−0.4 (4)	C59—C60—C61—O9	6.7 (4)
C24—C25—C26—C27	179.0 (2)	C59—C60—C61—C53	−177.0 (2)
C21—C26—C27—C19	2.6 (3)	O10—C62—C63—C64	2.3 (5)
C25—C26—C27—O4	3.9 (4)	O10—C62—C63—C68	−179.9 (3)
C21—C26—C27—O4	−176.7 (3)	C51—C62—C63—C64	−177.4 (2)
C25—C26—C27—C19	−176.8 (2)	C51—C62—C63—C68	0.4 (3)
C17—C28—C29—C34	−2.2 (3)	C62—C63—C64—C65	177.1 (3)
C17—C28—C29—C30	178.5 (2)	C68—C63—C64—C65	−0.5 (4)
O5—C28—C29—C30	−3.1 (4)	C62—C63—C68—C52	1.1 (3)
O5—C28—C29—C34	176.2 (3)	C62—C63—C68—C67	−177.9 (2)
C34—C29—C30—C31	−0.3 (4)	C64—C63—C68—C52	179.1 (2)
C28—C29—C34—C18	0.4 (3)	C64—C63—C68—C67	0.2 (4)
C28—C29—C34—C33	−178.8 (2)	C63—C64—C65—C66	0.4 (4)
C28—C29—C30—C31	179.0 (3)	C64—C65—C66—C67	0.1 (4)
C30—C29—C34—C18	179.8 (2)	C65—C66—C67—C68	−0.5 (4)
C30—C29—C34—C33	0.6 (4)	C66—C67—C68—C52	−178.3 (3)
C29—C30—C31—C32	−0.1 (4)	C66—C67—C68—C63	0.3 (4)

### Hydrogen-bond geometry (Å, º)

**Table:**

*D*—H···*A*	*D*—H	H···*A*	*D*···*A*	*D*—H···*A*
C12—H12···O3	0.93	2.37	2.693 (3)	100
C22—H22···O3	0.93	2.50	3.081 (3)	121
C24—H24···O10^i^	0.93	2.56	3.302 (3)	137
C30—H30···O5^ii^	0.93	2.60	3.507 (3)	166
C32—H32···O7^iii^	0.93	2.49	3.398 (3)	166
C33—H33···O4	0.93	2.32	3.087 (3)	139
C39—H39···O1^iv^	0.93	2.36	3.115 (3)	139
C46—H46···O8	0.93	2.40	2.728 (3)	101
C56—H56···O8	0.93	2.52	3.210 (3)	131
C58—H58···O7^v^	0.93	2.52	3.377 (3)	154
C67—H67···O9	0.93	2.36	3.117 (3)	139

Symmetry codes: (i) −*x*+2, −*y*, −*z*+1; (ii) −*x*, −*y*+1, −*z*+2; (iii) *x*−1, *y*, *z*; (iv) *x*+1, *y*−1, *z*; (v) −*x*+1, −*y*, −*z*+2.
